# In vivo photoprotection mechanisms observed from leaf spectral absorbance changes showing VIS–NIR slow-induced conformational pigment bed changes

**DOI:** 10.1007/s11120-019-00664-3

**Published:** 2019-09-20

**Authors:** Shari Van Wittenberghe, Luis Alonso, Zbyněk Malenovský, José Moreno

**Affiliations:** 1 Laboratory of Earth Observation, Image Processing Laboratory, University of Valencia, C/Catedrático José Beltrán, 2, 46980 Paterna, Valencia Spain; 2 Optics of Photosynthesis Laboratory, Institute for Atmospheric and Earth System Research/Forest Sciences, Faculty of Agriculture and Forestry, University of Helsinki, 00014 Helsinki, Finland; 3 Geography and Spatial Sciences, School of Technology, Environments and Design, University of Tasmania, Private Bag 76, Hobart, TAS 7001 Australia

**Keywords:** Absorbance shift, Absorbed photosynthetic active radiation (APAR), Controlled heat dissipation, Hyperspectral remote sensing, Non-photochemical quenching (NPQ), Passive chlorophyll a fluorescence, Pigment–protein dynamics

## Abstract

Regulated heat dissipation under excessive light comprises a complexity of mechanisms, whereby the supramolecular light-harvesting pigment–protein complex (LHC) shifts state from light harvesting towards heat dissipation, quenching the excess of photo-induced excitation energy in a non-photochemical way. Based on whole-leaf spectroscopy measuring upward and downward spectral radiance fluxes, we studied spectrally contiguous (hyperspectral) transient time series of absorbance *A*(*λ*,*t*) and passively induced chlorophyll fluorescence *F*(*λ*,*t*) dynamics of intact leaves in the visible and near-infrared wavelengths (VIS–NIR, 400–800 nm) after sudden strong natural-like illumination exposure. Besides light avoidance mechanism, we observed on absorbance signatures, calculated from simultaneous reflectance *R*(*λ*,*t*) and transmittance *T*(*λ*,*t*) measurements as *A*(*λ*,*t*) = 1 − *R*(*λ*,*t*) − T(*λ*,*t*), major dynamic events with specific onsets and kinetical behaviour. A consistent well-known fast carotenoid absorbance feature (500–570 nm) appears within the first seconds to minutes, seen from both the reflected (backscattered) and transmitted (forward scattered) radiance differences. Simultaneous fast Chl features are observed, either as an increased or decreased scattering behaviour during quick light adjustment consistent with re-organizations of the membrane. The carotenoid absorbance feature shows up simultaneously with a major *F* decrease and corresponds to the xanthophyll conversion, as quick response to the proton gradient build-up. After xanthophyll conversion (*t* = 3 min), a kinetically slower but major and smooth absorbance increase was occasionally observed from the transmitted radiance measurements as wide peaks in the green (~ 550 nm) and the near-infrared (~ 750 nm) wavelengths, involving no further *F* quenching. Surprisingly, in relation to the response to high light, this broad and consistent VIS–NIR feature indicates a slowly induced absorbance increase with a sigmoid kinetical behaviour. In analogy to sub-leaf-level observations, we suggest that this mechanism can be explained by a structure-induced low-energy-shifted energy redistribution involving both Car and Chl. These findings might pave the way towards a further non-invasive spectral investigation of antenna conformations and their relations with energy quenching at the intact leaf level, which is, in combination with *F* measurements, of a high importance for assessing plant photosynthesis in vivo and in addition from remote observations.

**Electronic supplementary material:**

The online version of this article (10.1007/s11120-019-00664-3) contains supplementary material, which is available to authorized users.

## Introduction

Photosynthetic light-harvesting complexes (Lhcs) are sophisticated multichromophoric assemblies used to regulate and concentrate photo-excitations under wide-ranging incident irradiances for delivery to the reaction centres (Scholes et al. 2011). To protect themselves and the reaction centres from a potentially harmful solar irradiance excess, several regulated photoprotection mechanisms are activated at different time scales at the level of these complexes, balancing out the given energy supply. This regulated lowering of the excitation pressure on the reaction centres decreases photochemical quenching as energy is non-photochemically quenched inside the leaves by various physical and chemical signals (Demmig-Adams and Adams III 1992). The fast or so-called energy-dependent quenching (qE) mechanism has been assigned to thermal deactivation of singlet excited chlorophyll (1Chl*) in the antenna of photosystem II (PSII), lowering the quantum yield of fluorescence (*F*) (Niyogi 1999). Although different mechanisms contribute to non-photochemical energy quenching (NPQ) in the short-term, qE is typically presented as the dominating form of controlled energy dissipation in leaves under most natural conditions (Holzwarth et al. 2009). The qE-quenching mechanism reacts to the prevailing light conditions within few minutes. As the proton gradient (ΔpH) is built-up, it triggers chemical conversions as an immediate photoprotection response to light excess (Johnson and Ruban 2014). Additionally, a slower-induced photoprotection mechanism, often called the ‘energy-independent’ photoprotection takes place on a slower timescale but is suggested to have a higher photoprotection impact compared to the faster energy or ΔpH-dependent response (Nilkens et al. 2010; Jahns and Holzwarth 2012; Lambrev et al. 2012). Currently, there is still debate on how and where the ΔpH-dependent and ΔpH-independent mechanisms take place in the Lhcs associated with the two photosystems (PSI and PSII), forming the supramolecular light-harvesting pigment–protein complexes (PSI-LHCI and PSII-LHCII). Further, questions still remain on how these mechanisms are triggered, and how they occur in vivo for different plant species as they are typically indirectly observed both in vivo and in vitro through fluorescence dynamics and accompanying shifts.

Last 30 years of NPQ research proved the central role of carotenoids (Cars), the important accessory pigments of the Lhcs (Demmig et al. 1987; Demmig-Adams et al. 1995). Cars absorb light in the blue-to-green spectral range (with small shifts depending on the molecule) and subsequently transfer the energy by multiple interactions between the excited energy states of the different antenna pigments (Young and Frank 1996). The xanthophyll cycles were shown to be involved in both direct and indirect ways and in both the ΔpH-dependent and the ΔpH-independent photoprotection mechanisms of the antenna, involving conformational changes in the Lhcs. Conformational changes are the structural changes within the Lhcs, which modulate the interaction between the chromophores (Ahn et al. 2008; Miloslavina et al. 2008; Müller et al. 2010; Jahns and Holzwarth 2012), i.e. the xanthophylls and chlorophyll, and the interaction between the chromophores and the protein scaffold itself (van Grondelle and Novoderezhkin 2006; Müh et al. 2010; Ostroumov et al. 2014). The well-known accumulation of zeaxanthin (Z), as a result of lumen acidification initiating the enzymatic conversion of the Car violaxanthin (V) to zeaxanthin (Z) via the intermediate antheraxanthin (A) in the VAZ xanthophyll cycle, is one of the short-term photoprotection mechanisms (seconds to minutes) (Bilger et al. 1989; Gamon et al. 1990; Peguero-Pina et al. 2013) that has been linked to several key processes triggering the NPQ mechanisms. After the chemical conversion into Z, which takes place in the lipid phase of the thylakoid membrane, the binding of Z to a Lhc induces a conformational change, which can be spectrally monitored by steady-state and time resolved *F* methods (Moya et al. 2001; Dall’Osto et al. 2005). Hence, the most common conformational changes observed and discussed in literature are the qE-dependent pigment-protein conformational changes that happen in the short term and drive the monomerization of the thylakoid membrane protein PS II subunit S PsbS (Deamer et al. 1967; Heber 1969; Bassi and Caffarri 2000; Moya et al. 2001; Ruban et al. 2007; Johnson et al. 2009a; Johnson and Ruban 2014; Krüger et al. 2014b). These PsbS-induced conformational changes involving aggregation and further a detachment of trimeric Lhc associated to PSII have been shown on a molecular basis (Correa-Galvis et al. 2016) and have been associated to low-energy (red-shifted) components in the absorption spectra of Lhcs (Johnson et al. 2009b). First zeaxanthin but later lutein was suggested to be involved in the further quick conformational changes, and held responsible for the triggering and maintaining of the structural protein folding (Croce et al. 1999; Bassi and Caffarri 2000). Despite these findings, the exact role of the different Cars in the antenna rearrangements remains controversial, mainly due to the lack of consensus on the molecular mechanisms in various conditions (Holt et al. 2004; Pascal et al. 2005; Jahns and Holzwarth 2012; Ruban et al. 2012). Overall, the Lhcs are shown to be effective probes of the quick conformational changes at the monomeric or trimeric unit (Johnson and Ruban 2009; Krüger et al. 2011, 2014a). However, little is known about the structural dynamics and transition between different conformational states available to the supramolecular PSII-LHCII and PSI-LHCI complexes being large ensembles of these Lhcs around their reaction centres.

Even slower induced organizational change of the Lhc, in which *Z* is implied to have an allosteric role, has also been raised (Dall’Osto et al. 2005; Lambrev et al. 2012). Based on *F* lifetime analysis it has been suggested that the Z-dependent conformational change takes place in the antenna proteins functionally connected to the PSII super-complex and to the PSII reaction centre (Dall’Osto et al. 2005; Holzwarth et al. 2009), even though the molecular basis is unclear and in vivo observations are lacking. Holzwarth et al. (2009) suggested hereby that by binding to Lhc proteins into multiple sites, the accumulated *Z* pool creates conditions for energy quenching by presumably modifying the hydrophobicity of the Lhc proteins, switching them into a further energy quenched state.

Several spectroscopy techniques, including transient absorption spectroscopy, fluorescence lifetime analysis, resonance Raman scattering, and circular dichroism, have observed and described in vitro significant reversible and quick reorganizations at the levels of (i) the ultrastructure of thylakoid membranes (Heber 1969; Krause 1973; Cseh et al. 2005), (ii) macro-organization of Lhcs within the membrane (Dall’Osto et al. 2005; Betterle et al. 2009), and (iii) purified Lhcs (Moya et al. 2001; Johnson and Ruban 2014). Although the relationship between NPQ and these different reorganizations remains to be further explored, provided evidence supports the idea that NPQ involves extensive reorganization of the Lhcs (Dall’Osto et al. 2005; Betterle et al. 2009; Garab 2016) opening energy dissipation channels that would trap the energy transferred amongst Chls (Krüger and van Grondelle 2017). However, as in vitro analysis of possible molecular mechanisms of NPQ are never performed on antenna complexes in their native environment (Krüger and van Grondelle 2017), the molecular basis and strengths of different energy-quenching mechanisms in vivo remains still unexplored (Holzwarth et al. 2009; Nilkens et al. 2010).

At the intact leaf level, absorbance changes related to NPQ have been often observed in parallel with additional scattering changes related to chloroplast movement (Bilger and Björkman 1990; Brugnoli and Björkman 1992; Kramer and Sacksteder 1998; Cazzaniga et al. 2013) or membrane rearrangements (Garab 2014), both complicating the disentanglement of the processes. These studies often focused on short-term (few minutes) changes observed commonly through a single wavelength, missing the dynamic behaviour of overlapping mechanisms. As an example, the spectral region of 500–570 nm has been exploited for the in vivo quantification of the VAZ xanthophyll cycle (Gamon et al. 1990; Evain et al. 2004), even though certain limitations have been noticed due to additional pigment bed changes (Ripullone et al. 2011). Hence, contiguous spectral data collections during sudden light transitions may allow for a more detailed observation of distinct spectral patterns during the light-induced reorganization of the antenna or entire chloroplasts. These may include a chromophore conversion (Gamon et al. 1990) and scattering changes due to chloroplast movement (Brugnoli and Björkman 1992), but also absorbance shifts due to changes in the local environment of the Lhcs (Johnson et al. 2009a), each displaying a specific spectral pattern. Due to this complexity, the specific contiguous spectral signatures linked to the various pigment–pigment interactions (including chemical conversions) and pigment–protein interactions (molecular rearrangements) are still poorly understood from in vivo observations and under natural-like illumination conditions. Distinguishing specific spectral signatures associated to distinct photo-avoidance and photoprotection processes would significantly advance the insights on the multichromophoric antenna dynamics upon light excess. Although optical leaf spectroradiometry dedicated to reflectance, transmittance and chlorophyll *F* measurements under natural solar or natural-like illumination is still underexplored, it has the potential to study such dynamics in vivo in SI energy units (Van Wittenberghe et al. 2015; Alonso et al. 2017; Aasen et al. 2019).

Therefore, the goal of this study is to explore how different photo-avoidance and photoprotection mechanisms can be detected and followed from contiguous spectral radiance measurements in the visible (VIS, 400–700 nm) and near-infrared (NIR, 700–800 nm) range. By analysing controlled dark-to-high-intensity light transients, our aim is to distinguish different spectral features from the information of bi-directional diffusively scattered and fluoresced light changes during the NPQ induction phase, if observable. With this, we intend to investigate the complexity of light interception and absorbance strategies by the antenna complexes of higher plants and the potential link with photoprotection dynamics in vivo, as this may serve as a further step in understanding vegetation dynamics from a remote sensing perspective.

## Materials and methods

### Leaf clip and dual spectroradiometer set-up

**Fig. 1:**
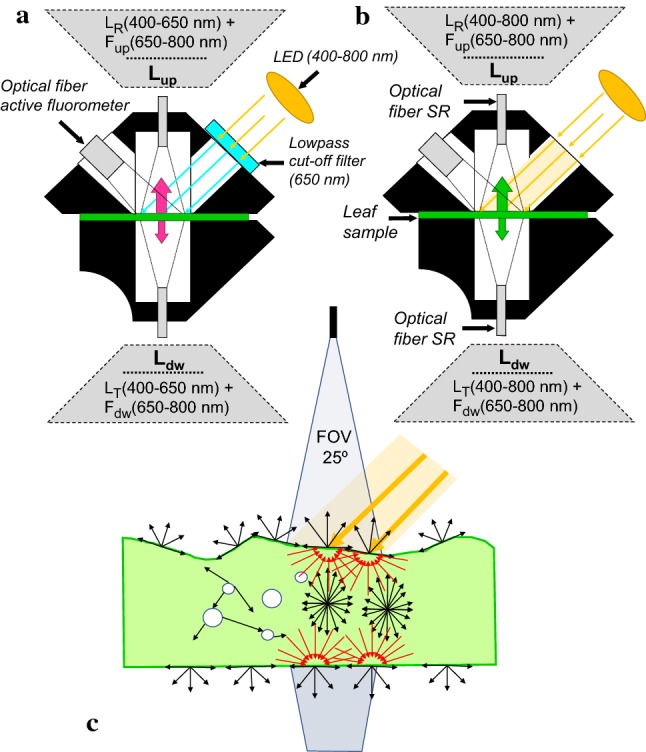
Leaf clip set-up for spectral contiguous upward and downward radiance flux (*L*_up_, *L*_dw_) measurements, with reflected and transmitted radiance (*L*_*R*_, *L*_*T*_) and passive fluorescence emission (*F*_up_, *F*_dw_) measurements under LED illumination using two spectroradiometers (SR), in combination with active *F* measurements. The set-up with corresponding measured fluxes is given with (**a**, protocol 1) and without (**b**, protocol 2) the 650-nm low-pass filter, measuring the radiance fluxes in the diffusively scattered light coming from the leaf surface diffusively scattered light (red arrows, **c**)

**Fig. 2:**
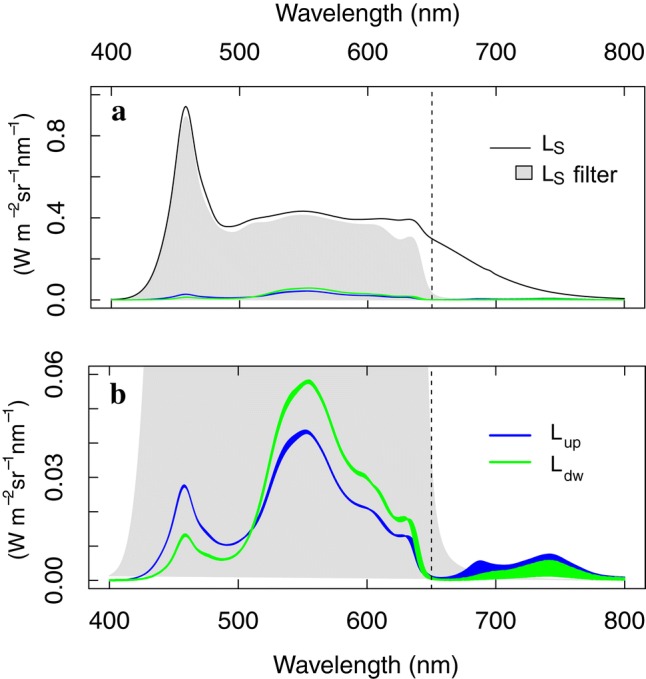
Surface radiance (*L*_*S*_(*λ*)) without (black line) and with (grey area as integrated over the PAR region) the 650-nm cut-off filter (dashed line) constant in time (**a**), and the upward and downward leaf dynamic radiance (*L*_up_(*λ*,* t*), *L*_dw_(*λ*, *t*)) measured during a dark-to-high light intensity transient using a sudden light intensity of 1311 µmol m^−2^ s^−1^ (296 W m^−2^) in the PAR region (**b**)

Hyperspectral signatures of leaf absorbance dynamics upon a high-intensity light (HL) adaptation were studied by measuring both upward and downward leaf diffusively scattered (reflected, transmitted), and diffusively emitted (fluoresced) radiance changes by means of two fibre optics, each attached to a spectroradiometer. Since all light not absorbed must be found as reflected (diffusively backscattered) or transmitted (diffusively forward scattered) light, while some absorbed light at a given wavelength may be emitted at longer wavelengths as fluorescence, a change in the overall hemispherical scattered light relates to a change in absorption or in fluorescence emission. We proposed a standardised laboratory set-up, in which single leaves attached to branches are clipped inside the custom-designed FluoWat leaf clip (Alonso et al. 2007; Van Wittenberghe et al. 2013) (Fig. 1) and mounted on a monopod ensuring a standardized portion of illumination entering the leaf clip chamber at an angle of 45°. A high-voltage LED (High Cri LED 10 W 17 V 3050–5900 K, Yuji International Co., Ltd, China), providing a broadband radiation spectrum between 400 and 780 nm with a peak in the blue part (Fig. 2a), was positioned towards the illumination port opening of the clip. To avoid overheating, the LED was glued on a heat sink and a small fan was attached for air-forced cooling. In front of the leaf-illumination opening, we attached a slider offering the three respective measuring options: (1) a dark cover, (2) a free opening, and (3) a high-performance OD4 low-pass filter (TechSpec, Edmund Optics GmbH, Germany) cutting off wavelengths > 650 nm. The latter option allows measuring changes in pure *F* emission between 650 and 800 nm (Figs. 1, 2b). The fibre optic of an ASD FieldSpec full-range (400–2500 nm) spectroradiometer (ASD Inc., Boulder, CO, USA) was inserted in the upper optical leaf clip opening (perpendicular direction to the leaf surface), while the second fibre optic of an ASD FieldSpec HandHeld 2 VNIR (325–1075 nm) spectroradiometer (ASD Inc., Boulder, CO, USA) was inserted in the bottom opening of leaf clip. This set-up allows simultaneous measurements of both upward and downward leaf radiance (*L*_up_(*λ*), *L*_dw_(*λ*), W m^−2^ sr^−1^ nm^−1^) measured respectively from the perpendicular directions to the adaxial and abaxial leaf side with a field of view (FOV) of 25° (Fig. 1c), a spectral accuracy of ± 1 nm and a resolution of < 3 nm. Leaves have both diffuse (near-Lambertian) and specular (non-Lambertian) characteristics, defining the hemispherical scattered light (Myneni and Ross 1991). Leaf transmittance, which only has the diffuse component, has a near-Lambertian distribution, while the distribution of reflected light depends on the incoming illumination angle. In our set-up, the 45° illumination angle and nadir viewing with a 25° FOV angle is used to minimize the effects of angle dependency and to obtain a near-Lambertian approximation for the reflected light. Despite this, an offset of 3.7 ± 1.0% in fAPAR, calculated for 30 leaves as the average of spectrally resolved absorbance in the region of photosynthetically active radiation (PAR, 400–700 nm) was found between our set-up and comparative integrating sphere measurements. This residual offset is mainly due to the lack of signal detection in the specular and neighbouring reflectance directions (i.e., specular component) of the incoming light by (1) the specific illumination and viewing geometry and (2) the selection of leaves with no extreme waxy or hairy surfaces. The diffuse character of the leaf is assumed to emanate primarily from the leaf interior, where each beam of light takes a unique path encountering different internal structures of varying geometric configurations and is scattered at each Refractive Index discontinuity (Kumar and Silva 1973). Hence, unlike measurements of homogenized in vitro suspensions (e.g. macromolecules in solution), where size of the particles might equal to the length of the measuring light wave and consequently produce an angularly anisotropic scattering, our in vivo optical measurements of leaves are angularly nearly isotropic, as the size of leaf-level scattering structures is larger than the measured wavelengths. In other words, the leaf surface behaves as a near-Lambertian scatterer, releasing prevailingly diffuse light originating from random multiple scattering within the ensemble of leaf interior elements. *L*_up_(*λ*) and *L*_dw_(*λ*) are, respectively, composed of the reflected and transmitted radiance (*L*_*R*_(*λ*), *L*_*T*_(*λ*), W m^−2^ sr^−1^ nm^−1^) in the 400–800 nm range and the upward and downward Chl a *F* emission (*F*_up_(λ) and *F*_dw_(*λ*), W m^−2^ sr^−1^ nm^−1^) in the 650–800 nm region (Fig. 1a):

$$L_{\text{up}} \left( { 400 - 800{\text{ nm}}} \right) = L_{R} \left( { 400 - 800{\text{ nm}}} \right) + F_{\text{up}} \left( { 6 50 - 800{\text{ nm}}} \right)$$

$$L_{\text{dw}} \left( { 400 - 800{\text{ nm}}} \right) = L_{\text{T}} \left( { 400 - 800{\text{ nm}}} \right) + F_{\text{dw}} \left( { 6 50 - 800{\text{ nm}}} \right)$$

The measuring fibre of an active fluorometer (PAM-2000, Walz GmbH, Effeltrich, Germany) can also be inserted in an opening opposite of the illumination opening (Fig. 1). Branches of *Morus alba* L. (White mulberry) and *Juglans regia* L. (Walnut) were freshly collected in June and July 2017 from the south-facing side of the trees, grown under a Mediterranean climate. Sun-grown leaves were chosen to select leaves optimized for an increased photoprotection capacity to excessive light. The branches were, immediately after cutting, submerged into water, transported to the lab, and kept in a dark place for 2 h. Single-attached leaves were placed inside the leaf clip, while the dark cover slid was placed in front of the illumination opening. Both spectroradiometers were set to measure continuously single spectra with an integration time of 136 (FieldSpec) or 272 (HandHeld) ms. The reason for different integration times was a faster data collection of the HandHeld and our intention of collecting simultaneous *L*_up_ and *L*_dw_ spectra. Despite our efforts, the instruments’ acquisitions could not be perfectly synchronized, resulting in need of proximate synchronization of both spectral transient series during data processing.

### Dark-to-high-intensity light-transient protocols

A leaf under a saturating HL treatment typically converts V to A and to Z within approx. 10 min of high-intensity light (Bilger et al. 1989; Johnson et al. 2009a), although even shorter kinetical conversion steady-states have been measured at 3–5 min in vivo (Bilger et al. 1989; Peguero-Pina et al. 2013). In contrast, longer conversion steady-states around 60 min have been seen for different antenna subcomplexes in vitro (Heyde and Jahns 1998). A mathematical kinetic model description of VAZ conversion, originally applied for a system of liposome membranes (Latowski et al. 2000), has however, been functionally applied to the quick NPQ-transient phase, i.e. 0–2 min after dark to light exposure (D’Haese et al. 2004), kinetically indicating the quick VAZ conversion in vivo in the timescale of few minutes. Therefore, dark-to-HL transients of 10 min were applied to the leaves with an incoming LED illumination of approx. 270 W m^−2^ (1200 µmol photons m^−2^ s^−1^) in the PAR region. Two different protocols were used to study the spectrally contiguous transient scattering and absorption dynamics. In the first protocol (protocol 1), the 650-nm cut-off low-pass filter was slid in front of the illumination opening, allowing to observe only *F* emission in the 650–800 nm region (Fig. 1a). It is important to note that an internal photon scattering, taking place between the leaf and the filter, could potentially increase the radiance measured in the 620–650 region. This possible artefact is, however small, if being significant, due to the black and light-absorbing interior of the clip. Other possible artefacts due to the leaf clip design (e.g. filter transmission), the spectroradiometer (e.g. dark noise correction) and the set-up (e.g. stray light) were accounted for or diminished (Supplementary material S1). Any remaining instrumental artefact, even if small, can be considered constant in time and thus would not alter the variations in *L*_up_ and *L*_dw_. Besides passive radiative measurements under LED illumination, additional active pulse-amplitude-mode (PAM) red-saturating flashes (400 ms, 7000 μmol photons m^−2^ s^−1^) were applied to derive the PAM-NPQ parameter based on broadband (710–850 nm) relative *F* changes as: (Fm–Fm′)/Fm′, where Fm′ is the maximum *F* at each saturation pulse every 30 s. After a dark adaptation of a minimum 2 h, a saturation flash was given to determine Fo and Fm, respectively, the minimal and maximal fluorescence signal. One minute later, the dark-to-HL transient was executed, while *L*_up_ and *L*_dw_ spectra were continuously recorded (Fig. 2b).

During the second protocol (protocol 2), the 10-min dark-to-HL transient was performed without low-pass filter in front of the illumination opening (Fig. 1b). Executing this protocol, *L*_*R*_ and *L*_*T*_ were measured over the VIS–NIR region (400–800 nm) and mixed with, respectively, *F*_up_ and *F*_dw_ in the 650–800 nm region. Table 1 overviews the measured radiance fluxes and ratios calculated during the transient, implying a time dimension. After each transient protocol a Lambertian white spectral reference (Spectralon, Labsphere Inc., North Sutton, USA) was placed inside the leaf clip to measure the radiance arriving to the surface (*L*_S_(*λ*,*t*), W m^−2^ sr^−1^ nm^−1^) in the LED spectral region which was tested to be stable through time (Fig. 2a). Changes in reflected and transmitted radiance (Δ*L*_R_(*λ*, Δ*t*) and Δ*L*_T_(*λ*, Δ*t*), W m^−2^ sr^−1^ nm^−1^) are given a Δ*t* = *t *− *t*_0_, *t*_0_ being the reference time chosen. Simultaneously *F* emission changes (Δ*F*_up_(*λ*, Δ*t*) and Δ*F*_dw_(*λ*, Δ*t*), W m^−2^ sr^−1^ nm^−1^) may occur. Hence,

$$\Delta L_{\text{up}} \left( { 400 - 800{\text{ nm}}} \right) = \Delta L_{\text{R}} \left( { 400 - 800{\text{ nm}}} \right) + \Delta F_{\text{up}} \left( { 6 50 - 800{\text{ nm}}} \right)$$

$$\Delta L_{\text{dw}} \left( { 400 - 800{\text{ nm}}} \right) = \Delta L_{T} \left( { 400 - 800{\text{ nm}}} \right) + \Delta F_{\text{dw}} \left( { 6 50 - 800{\text{ nm}}} \right)$$

$${\text{And }}\Delta {\text{L}}_{\text{tot}} \left( { 400 - 800{\text{ nm}}} \right) = \Delta {\text{L}}_{\text{up}} \left( { 400 - 800{\text{ nm}}} \right) + \Delta {\text{L}}_{\text{dw}} \left( { 400 - 800{\text{ nm}}} \right)$$

To investigate the leaf spectral dynamics during the induction phase of photoprotection, we calculated the spectral differences for different quenching transient phases by subtracting the first radiance spectrum from each consecutive transient radiance spectra of each phase. In the 400-650 nm spectral region where no Chl *F* emission nor *F* emission change from any other component upon photoprotection dynamics occurs (Buschmann et al. 2000), reflectance and transmittance changes (Δ*R* and Δ*T*) were calculated for Δ*t* = *t* − *t*_0_ by dividing Δ*L*_R_ and Δ*L*_T_ with corresponding *L*_S_ and used to derive the leaf absorbance change (Δ*A*):

$$\Delta R\left( {\lambda , \, \Delta t} \right) = {{\left( {L_{R} \left( {\lambda , \, t} \right) - \, L_{R} \left( {\lambda , \, t_{0} } \right)} \right)} \mathord{\left/ {\vphantom {{\left( {L_{R} \left( {\lambda , \, t} \right) - \, L_{R} \left( {\lambda , \, t_{0} } \right)} \right)} {L_{S} \left( \lambda \right)}}} \right. \kern-0pt} {L_{S} \left( \lambda \right)}}$$

$$\Delta T\left( {\lambda , \, \Delta t} \right) = {{\left( {L_{T} \left( {\lambda , \, t} \right) - \, L_{T} \left( {\lambda , \, t_{0} } \right)} \right)} \mathord{\left/ {\vphantom {{\left( {L_{T} \left( {\lambda , \, t} \right) - \, L_{T} \left( {\lambda , \, t_{0} } \right)} \right)} {L_{S} \left( \lambda \right)}}} \right. \kern-0pt} {L_{S} \left( \lambda \right)}}$$

Variations in surface feature irregularities with a size similar to the wavelength (leading to variable at-surface Mie scattering) are considered negligent compared to the variations in leaf-internal surfaces of varying geometric configurations inside the leaf. Hence, at-surface spectral changes are determined by the diffuse at-surface component rising from the random multiple scattering between the internal structures. This random and angularly independent character at leaf level is different from the possible variation in in-path Mie scattering during structural changes which may be considerable and detectable for single chloroplasts or other spherical leaf subsystems measured in solution due to significant horizontal light transport or strong light diffusion in the media (Latimer and Pyle 1972; Heirwegh et al. 1987). Nevertheless, although the at-leaf-surface diffuse component may be quasi-angularly anisotropic for each side, it is important to take into account the diffuse component of both adaxial and abaxial sides to account for the angular effects at leaf surface level determined by the overall internal scattering between geometrical arrangements and structures. Hence, our set-up results in an emanating approx. hemispherical leaf surface-leaving radiance insensitive to the in-path changes after multiple scattering (Fig. 1c), where total absorbance changes (Δ*A*) are only given by specific absorption changes. Consequently, in the absence of *F*, absorbance changes are estimated as the negative sum of changes in diffusively scattered illumination:

$$\Delta A\left( \lambda \right) = - \, \left( {\Delta R\left( \lambda \right) + \Delta T\left( \lambda \right)} \right),$$

whereby Δ*A* is given by the specific absorbance changes of the elements. Finally, the fluorescence yield (FY(650–850 nm), rel.) was calculated as the ratio of the spectrally integrated *F* emission (650–850 nm) and the spectrally integrated absorbed PAR (400–700 nm) for, respectively, the upward, the downward and the total component (FY_up_, FY_dw_ and FY_tot_) (Van Wittenberghe et al. 2013). Spectral dynamics during the transients were investigated by analysing the maximal Δ*R* and Δ*T* and their shifts in time. Two spectral shifts of maximal Δ*R* and Δ*T* wavelength were used to analyse and to distinguish different absorption features during the light transients. All spectral processing and artwork were performed in the R software version 3.3.1 (www.r-project.org), making use of the asdreader library to import the spectral data. No spectral filters were used before data processing, only a local regression smoothing was occasionally applied for visual presentations.

**Table 1:** Measured and calculated radiance fluxes and ratios with a wavelength (*λ*) and time (*t*) dimension for the leaf clip with dual spectroradiometer set-up

Parameter	Definition
*L*(*λ*, *t, ω*), W m^−2^ sr^−1^ nm^−1^	Spectral radiance flux emitted, reflected, transmitted or received by a given surface, measured for a given angular direction
*L*_up_(*λ*, *t*), W m^−2^ sr^−1^ nm^−1^	Upward *L*, perpendicularly measured to the adaxial leaf surface or WR surface which is illuminated
*L*_dw_(*λ*,*t*), W m^−2^ sr^−1^ nm^−1^	Downward *L*, perpendicularly measured to the abaxial leaf surface or WR surface of which is illuminated from the opposite site
*L*_tot_(*λ*,*t*), W m^−2^ sr^−1^ nm^−1^	Total *L*, *L*_up_ + *L*_dw_
*L*_S_(*λ*,*t*), W m^−2^ sr^−1^ nm^−1^	Radiance received by the surface, measured as *L*_tot_ from the WR panel
*L*_*R*_(*λ*, *t*), W m^−2^ sr^−1^ nm^−1^	*L*_up_ component, diffusively scattered from the leaf surface (and its interior) without fluorescence
*L*_T_(*λ*, *t*), W m^−2^ sr^−1^ nm^−1^	*L*_dw_ component, diffusively scattered from the leaf surface (and its interior) without fluorescence
*R*(*λ*, *t*), –	Reflectance, *L*_R_(*λ*,*t*)/*L*_*S*_(*λ*)
*T*(*λ*, *t*), –	Transmittance, *L*_*T*_(*λ*,*t*)/*L*_*S*_(*λ*)
*A*(*λ*, *t*), –	Absorbance, *A*(*λ, t*) = *1 *− *R(λ,t*)− *T*(*λ*,*t*)
*F*_up_(*λ*, *t*), W m^−2^ sr^−1^ nm^−1^	*L*_up_ component fluoresced from the leaf surface, given illumination to the adaxial side
*F*_dw_(*λ*,*t*), W m^−2^ sr^−1^ nm^−1^	*L*_dw_ component fluoresced from the leaf surface, given illumination to the adaxial side
*F*_tot_(*λ*, *t*), W m^−2^ sr^−1^ nm^−1^	Spectral radiant flux fluoresced from the abaxial and abaxial leaf surface, given illumination to the adaxial side, *F*_up_ + *F*_dw_
FY (*t*), –	Fluorescence yield, wavelength-integrated fluoresced flux (up, dw or tot) divided by *L*_*S*_ in the PAR (400–700 nm)

## Results

### Scattering versus absorbance increases

**Fig. 3:**
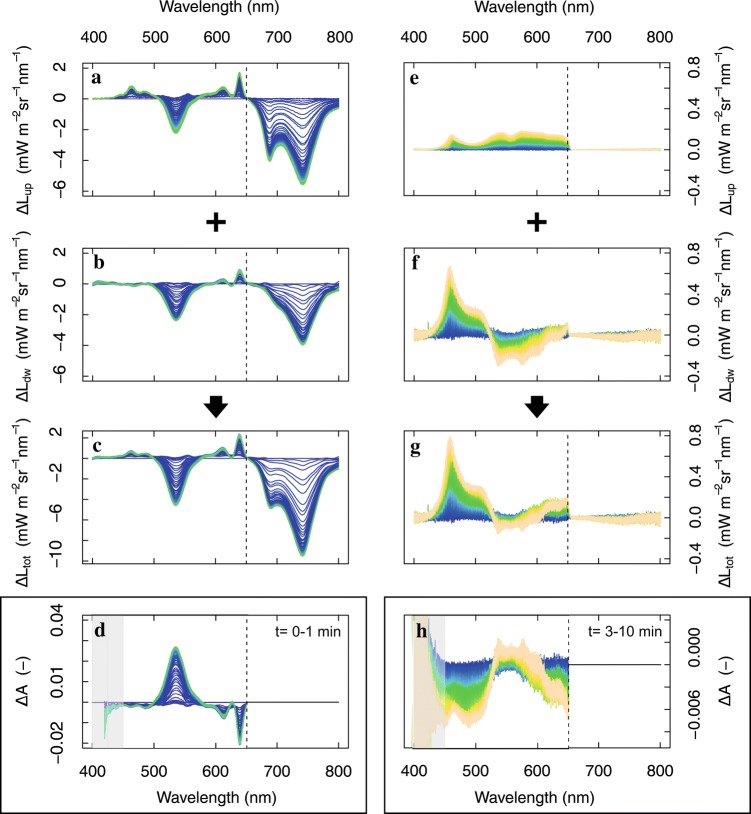
Examples of quick (left column, **a**–**d**) and slow (right column, **e**–**h**) radiative changes (Δ*L*_up_, Δ*L*_dw_, Δ*L*_tot_, mW m^−2^ sr^−1^ nm^−1^) from two different leaves measured using a 650-nm cut-off filter (protocol 1), showing quick narrow overlapping Δ*A* features with dominant 500–570 nm feature (**d**) and slow broad decreasing Δ*A* features suggesting a possible slow chloroplast motion (grey area marks low irradiance range)

A sudden interception of high-intensity illumination by the antenna causes the co-occurrence of several internal adjustment effects. Measured radiance changes are hereby wavelength-dependent and process-dependent, and may result from (1) a modified interception behaviour, e.g. by reorganizations at different hierarchical levels, i.e. at the level of thylakoid membrane or at the level of the entire chloroplast, and/or from (2) a modified absorbance behaviour, i.e. from molecular conversions or modified interactions between the chromophores. Both in the short (*t* = 0–1 min) and longer response (*t* = 1–10 min), several of these processes occur with distinct spectral responses (Fig. 3). Light avoidance is typically seen as an increased overall scattering to lower photon interception by the antenna. A case is shown within the short-term, fast narrow-featured scattering increases, in combination with scattering decreases (Fig. 3, left), which will be further discussed in the following section. In the longer response (*t* = 1–10 min), we occasionally observed stronger scattering increases, which were not necessarily conclusive from only forward or backscattered radiance changes, i.e. acting in the opposite direction in the green spectral region (Fig. 3b). This can be understood from the fact that scattering will depend on the overall change in orientation of the absorbing elements. When combining both upward and downward-scattered light, the total scattering increase from Δ*L*_tot_ shows the action spectra consistent with chloroplast motion (Brugnoli and Björkman 1992), being a rather slow process with a marked absorbance peak around 450 nm and shoulder around 475 nm (Inoue and Shibata 1973). A slight decrease in *F* emission consistent with a lower chloroplast absorbance is simultaneously observed. In further results, we show examples in which slow chloroplast motion was only slightly or not observed at all, as it is not part of the regulated photoprotection and it complicates the detection of other features, which may be of interest.

### Fast *F*-coupled spectral dynamics during NPQ induction (protocol 1)

**Fig. 4:**
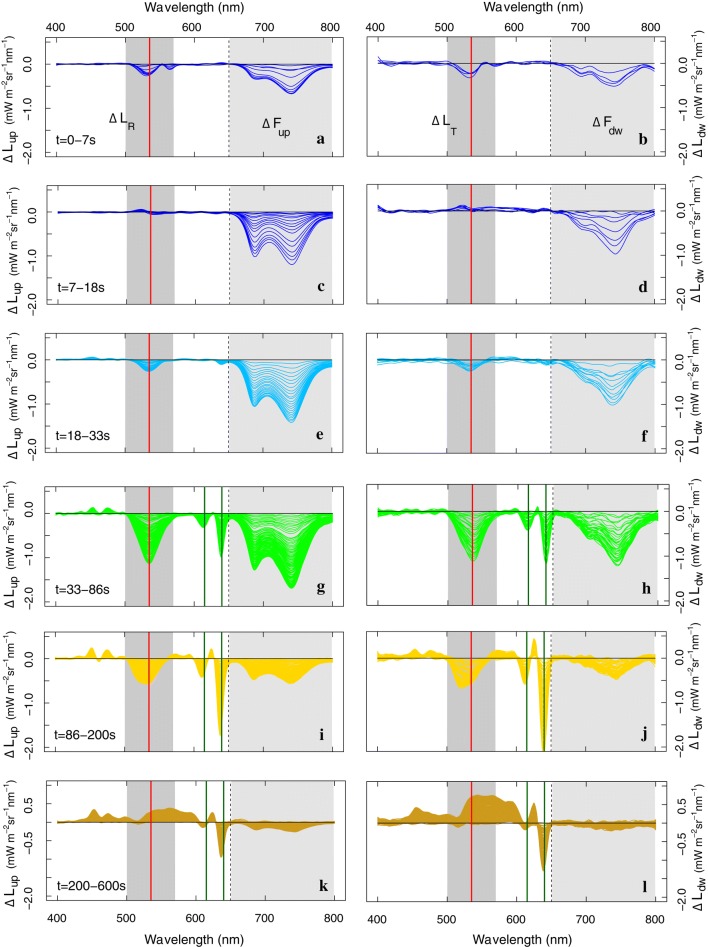
Upward (left column) and downward (right column) radiative change (Δ*L*_up_ and Δ*L*_dw_, mW m^−2^ sr^−1^ nm^−1^), with reflected and transmitted changes (Δ*L*_R_ and Δ*L*_*T*_) in the VIS (400–650 nm) and *F* emission changes in the red and far-red (650–800 nm, light grey shade) of a *M. alba* leaf for different photoprotection phases during the 10-min light transient (1200 µmol m^−2^ s^−1^), when using a 650-nm cut-off filter (dashed line). The reference spectrum is the first spectrum of each phase, respectively, showing only radiative changes for each specified period. The highly dynamic spectral region of 500–570 nm is marked in a dark grey shade. Major absorption wavelengths are marked as follows: red (531 nm) and dark green (615 and 645 nm)

**Fig. 5:**
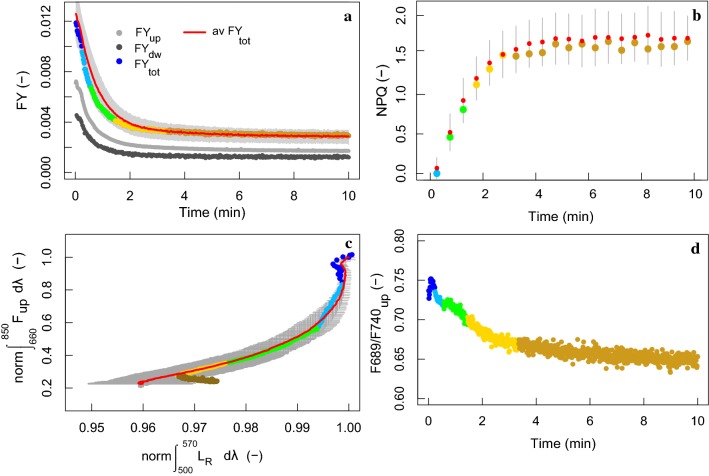
Upward, downward and total fluorescence yield (FY, rel.). (**a**), non-photochemical fluorescence quenching (PAM-*NPQ*) (**b**), normalized difference of the integrated upward fluorescence (*F*_up_, 660–850 nm) in respect to *t* = 0 min versus normalized difference of the integrated reflected radiance (*L*_*R*_, 500–570 nm) in respect to *t* = 0 min (**c**), and upward fluorescence peak ratio F689/F740 (**d**) presented for the 10-min dark-to-high-intensity light transient given to the *M. alba* leaf presented in Fig. 3. Colours indicate the different phases during the transient as presented in Fig. 3, which are distinguished based on the variable kinetic behaviour of both *F*_up_ (660–850 nm) and *L*_*R*_ changes (500–570 nm). Additional average (in red) and standard deviation (in grey) values are computed for five dark-adapted leaves measured under identical illumination conditions (1200 ± 65 µmol m^−2^ s^−1^) (**a**–**c**)

Multiple fast narrow-featured spectral changes appear when applying the dark-to-HL protocol with the filter placed in front of the illumination opening, in parallel to immediate strong *F* quenching as seen previously in Fig. 3 (left panel). Figure 4 shows the distinct spectral behaviour for separate Δ*t* phases during the transient of a single *M. alba* leaf, while Fig. 5 shows the overall behaviour of FY, PAM-NPQ, spectrally integrated *F*_up_ versus spectrally integrated *L*_*R*_ and for five leaves. Generally, a prompt decrease of upward and downward spectrally resolved fluorescence (Fig. 4a–d) and spectrally integrated FY is observed (Fig. 5a), followed by a much slower *F* quenching until the end of the 10-min transient. Changes in FY_up_ are also reflected by the increase of the PAM-NPQ parameter, which saturates after approx. 4 min after sudden exposure to HL illumination (Fig. 5b). During the adaptation to HL, several changes in reflected and transmitted radiance were observed within the VIS (400–650 nm), some of them showing consistent decreases in scattering, while others showing opposite behaviour depending on the case (Figs. 3–left vs. 4). The largest Δ*L*_*R*_ and Δ*L*_*T*_ right upon light exposure were observed in the 500–570 nm region, showing consistent decreases for all observations. To illustrate the trend of this feature, the integral of the Δ*L* in this region was calculated as the difference of the radiance with the first spectrum (*t* = 0 min), and normalized to the maximal difference, i.e., the integral of Δ*L* at *t* = 10 min. For comparison with the co-occurring *F* quenching, it was plotted against the co-occurring *F*_up_ emission change in the 660–850 nm region (Fig. 5c). Several distinct response phases were distinguished based on the variable kinetic behaviours of both integrated *F*_up_ (660–850 nm) and *L*_R_ changes (500–570 nm) and presented in Fig. 4. Further, we observed a non-uniform *F* quenching across the full spectral *F* range. Figure 5d additionally demonstrates different phases of the red (687 nm) to far-red (740 nm) *F* ratio evolution. A first small rapid rise of the F687/F740 ratio resulted in a local peak, which was followed by a steep decrease during the first 3 min and a mild, but steady, decrease in the following minutes.

Primary *F* quenching involved only minor radiative changes in the VIS wavelengths (Fig. 4a–d). Nevertheless, the on-set of *F* quenching took place rapidly in a period of seconds to 1 minute. An instant double-peak absorbance in the 500–570 nm region appeared quickly after illumination (Fig. 4a, b), followed by a *F* quenching without any VIS spectral change (Figs. 4c, d, 5c). Subsequently, further leaf absorption changes in the 500–570 nm region re-occurred, seen from Δ*L*_*R*_ and Δ*L*_*T*_ decreases (Fig. 4e–h). Decrease in normalized radiance (500–570 nm) shows two differently steep quenching slopes (Fig. 5c), i.e. initially relatively small 500–570 nm radiative changes with a strong *F* quenching, followed by a stronger absorption in the 500–570 nm wavelengths accompanied by a strong *F* quenching. Therefore, the two quenching phases were separated as presented in Fig. 4e–h. The initial radiative 500–570 nm changes, involving a high *F* quenching, were characterized by a bell-shaped curve centred around 535 nm (Fig. 4e–f), while the following stronger changes extend slightly beyond 570 nm and accompanied by narrow-band features in the red region, with peaks located at 615 and 645 nm, and minor features in the blue region (Fig. 4g, h). The red features became stronger during the next phase, while additional VIS features seem to overlap the 500–570 nm feature (Fig. 4i, j). Ultimately, spectral changes during the last phase (*t* = 200–600 s) showed a negative-to-positive twist in 520–600 nm absorbance values and weakening of the red features, while the overall scattering in the VIS increased (Fig. 4k, l).

**Fig. 6:**
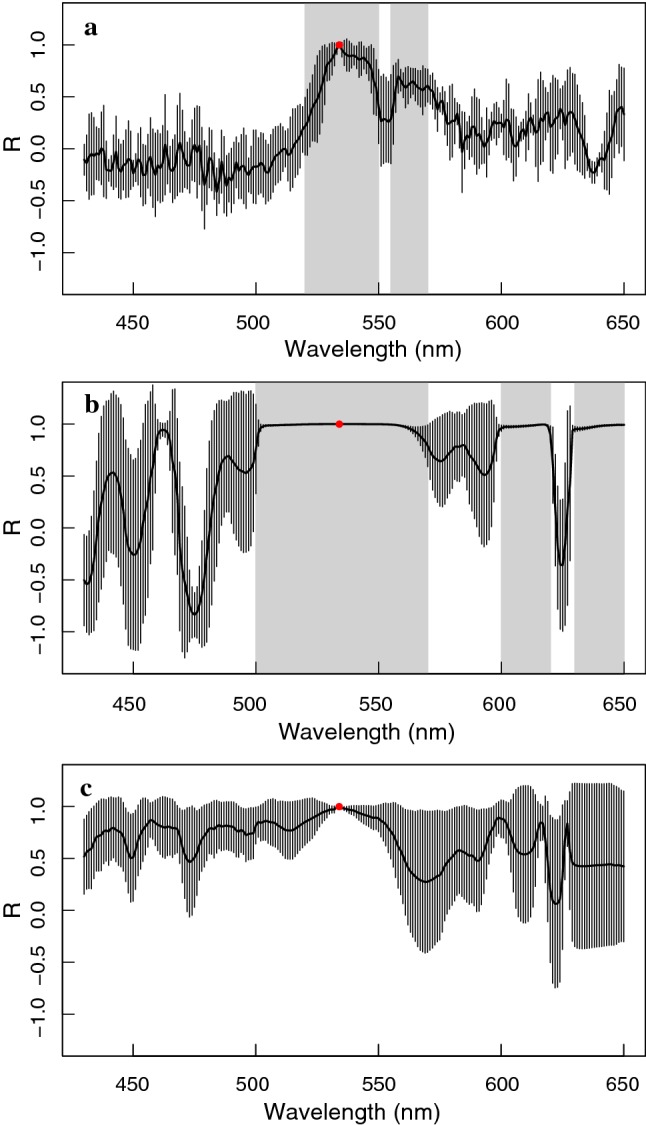
Correlation coefficient (*R*) ± standard deviation between Δ*R* at 535 nm (red point) with the reflectance wavelength changes (430–650 nm) during *t* = 0–20 s (**a**), *t* = 20–180 s (**b**), and *t* = 180–600 s (**c**) for five *M. alba* leaves exposed to a high-intensity illumination (1162 ± 30 µmol m^−2^ s^−1^); stand-out regions of high correlation are indicated with grey areas in **a**, **b**

Although some variable scattering behaviour could be detected in spectral regions where absorption of several foliar pigments overlaps (i.e. 400–500 nm), the dynamic changes in the region where photoprotection-related spectral features occur (i.e. 500–570 nm) were found to be time-consistent in the first 3 minutes upon illumination for five different leaves tested (Fig. 5c). This can be seen across different examined plant species tested here and in other works (Van Wittenberghe et al. 2018; Vilfan et al. 2018). Reflectance and transmittance changes of the shown feature occurred in equal magnitude pointing to a true absorbance increase. Other consecutive or simultaneous minor scattering changes within the full VIS spectrum (Fig. 4k, l) were not every time strongly or consistently detected (Figs. 3-left vs. 4). Hence, additional fast heterogeneous spectral changes occurred. Taking the reflectance changes at 535 nm as reference wavelength, only the changes in the 500–570 nm region resulted highly correlated during *t* = 0–20 s (Fig. 6a), while changes the first minutes hereafter (*t* = 20–180 s) were strongly correlated with the entire 500–570 nm region as well as with the 600–620 and 630–650 nm regions (Fig. 6b). After 3 min, when the 500–570 nm feature has terminated its activity, various spectral changes in the 430–650 nm region occur, showing further complex behaviour during light adaptation (Fig. 6c).

### Slow *F*-uncoupled spectral dynamics during NPQ induction (protocol 1)

**Fig. 7:**
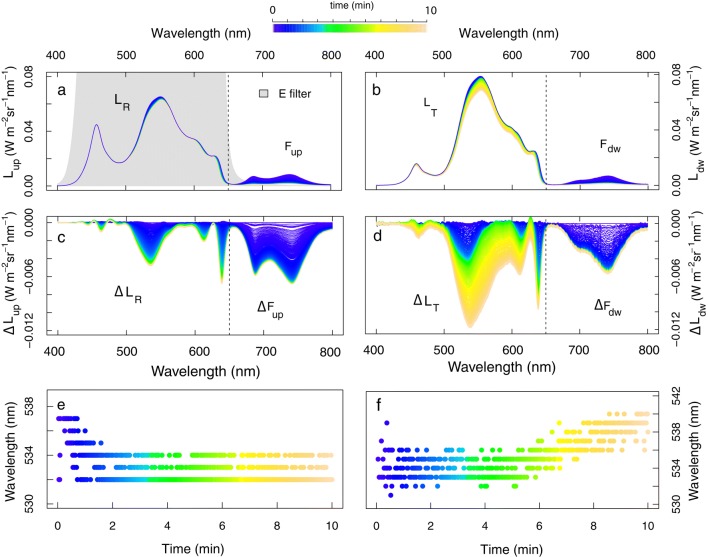
Upward and downward radiance (*L*_up_ and *L*_dw_) and *F* emission (*F*_up_, *F*_dw_) of a dark-adapted *M. alba* leaf measured upon sudden high-intensity LED illumination (1311 µmol m^−2^ s^−1^ or 296 W m^−2^ in the PAR region, partly illustrated as grey shade) during a 10-min transient and the filter protocol (**a**, **b**). Transient changes are displayed for *L* in the VIS (400–620 nm) and for *F* in the fluorescence range (650–800 nm), with the spectrum at *t* = 0 min taken as the reference spectrum (**c**, **d**). The wavelengths of the maximal Δ*R* and Δ*T* change within the 430–600 nm range during the transient are plotted in **e** and **f**

For both species, several leaves subjected to the dark-to-HL transient showed more pronounced scattering changes, in comparison to the results presented in the sect. “Scattering versus absorbance increases”. Figure 7 presents radiative evolution for a *M. alba* leaf upon a 10-min transient. It shows the upward and downward radiance spectra (a–b) and the respective changes (Δ*L*_up_ and Δ*L*_dw_), using the spectrum at *t* = 0 min as the reference (c–d). Fast radiative changes in the green (500–570 nm) and red (615 nm) wavelengths, which were previously shown in Fig. 4, were observed from the upward and downward signal during the first 2 min after illumination (blue-drawn spectra). Simultaneously, *F* was rapidly quenched. Further significant radiative changes were observed in *L*_*T*_ from *t* = 5 min onward (lime-drawn spectra), following the prior fast changes (Fig. 7d). Compared to the previously observed green absorbance change, this consecutive and slower decrease in scattering, only seen from decrease of Δ*T*, is stronger and, moreover, taking place across the full VIS spectral range (until 650 nm where illumination was cut-off). Surprisingly, a strong total decrease in scattering is observed here, which intuitively contradicts with a light-avoidance strategy. i.e. as given by chloroplast motion (Fig. 3right). No further simultaneous *F* quenching was measured during these changes after *t* = 2 min. The evolution of the wavelength with a maximal change during the light adaptation in the 430–600 nm region is shown for Δ*R* (Fig. 7e) and for Δ*T* (Fig. 7f). The Δ*R* peak maximum shifts slightly from 537 to 533 nm during the first 2 min and it remains stable hereafter. Δ*T* showed a clear peak wavelength shift to longer wavelengths during *t* = 5–10 min, i.e. from approx. 534 to 539 nm, indicating an additional absorbance feature appearance with different maximum location.

**Fig. 8:**
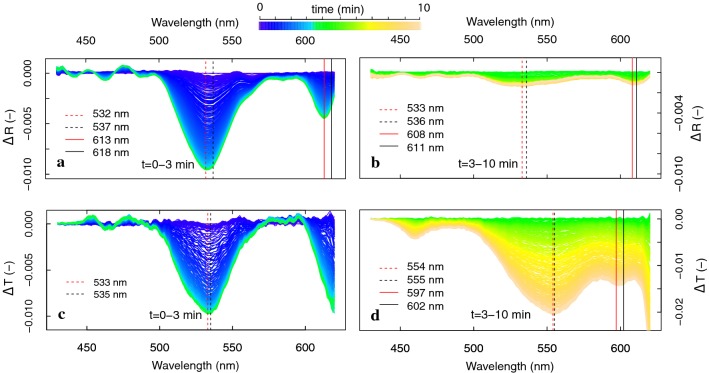
Reflectance and transmittance change (Δ*R* and Δ*T*), separated into the fast (*t *= 0–3 min) and slow (*t* = 3–10 min) changes during photoprotection reactions of a dark-adapted *M. alba* leaf exposed to a high irradiance (1311 µmol m^−2^ s^−1^). Location of peak maxima are given for the start (black lines) and the end (red lines) of the transient phases

**Fig. 9:**
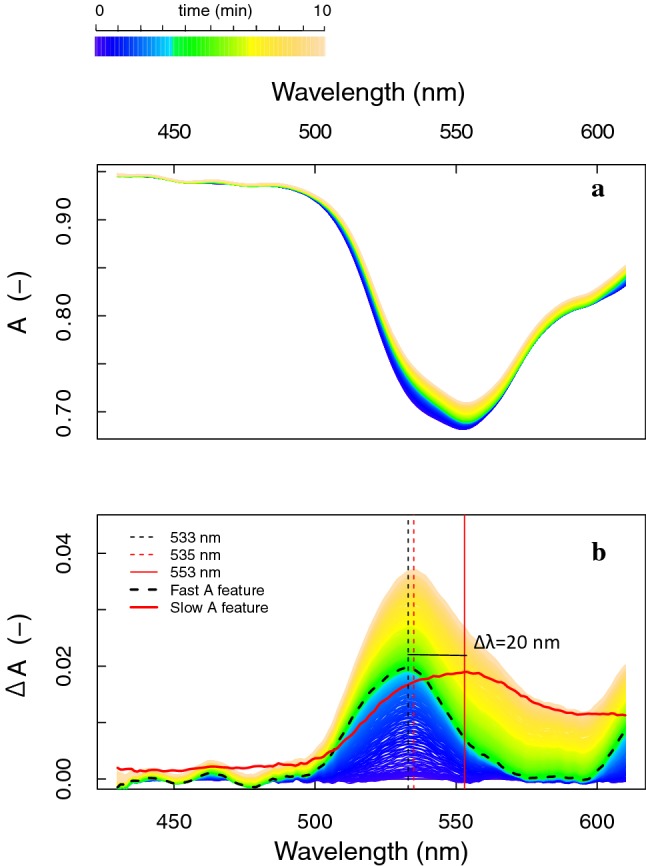
Absorbance (*A*, –) (**a**) and absorbance change (Δ*A*, –) (**b**) for a spectral interval of 430-620 nm during photoprotection of a dark-adapted *M. alba* leaf exposed to a high irradiance (1311 µmol m^−2^ s^−1^); The fast (*t* = 0–3 min) and slow (*t* = 3–10 min) absorbance features changes are displayed as the bold dashed black and the solid red line, respectively. The absorbance maxima at the start (black dashed) and at the end (red dashed) of the 10-min transient are plotted together with the absorbance peak of the slow component (red solid)

To separate the *F*-coupled and *F*-uncoupled absorbance features observed from Δ*R* and Δ*T*, a new reference spectrum was taken at *t* = 3 min for calculation of changes in both *R* and *T*, i.e. before the maximum peak shifts shown in Fig. 7g, h, and consequently subtracted from the consecutive dynamic radiance signal. The fast or ΔpH-dependent absorbance changes (*t* = 0–3 min) were almost equally represented in shape and magnitude of Δ*R* and Δ*T* (Fig. 8a, c). A minor absorbance peak shift towards shorter wavelengths (Δ*R*: 537 to 532 nm; Δ*T*: 535 to 533 nm) indicates the existence of more than one A feature during these early fast changes. Slow absorbance changes (*t* = 3–10 min) appeared to be minor in terms of the Δ*R*, but clearly observable from the Δ*T* spectra (Fig. 8b, d). Figure 8d shows an additional Δ*T* decrease of 2% during this period. The maximum Δ*T* decrease located at 554–555 nm remained stable, suggesting existence of a single absorbance feature at that wavelength during the given transient period. Due to the use of a cut-off filter, the full red and near-infrared spectral changes until 800 nm could not be shown. Calculation of the absorbance (*A*) and the absorbance changes (Δ*A*) and their separation into fast and slow absorbance features for the 430–620 nm region measured during the 10-min transient period are shown in Fig. 9. The fast Δ*A* showed a pronounced absorption feature in the green wavelengths (maximum at 533 nm, vertical dotted line), whereas the slow Δ*A* revealed a broadened absorbance peak shifted towards longer wavelengths, with a maximum at 553 nm (vertical red solid line). Both peaks showed an equal 2% increase in absorbance at their respective peak wavelength, showing a Δ*λ* = 20 nm. Moreover, it seems that the slow Δ*A* extend beyond 620 nm.

### Fast and slowly induced absorbance features in the VIS and NIR (protocol 2)

**Fig. 10:**
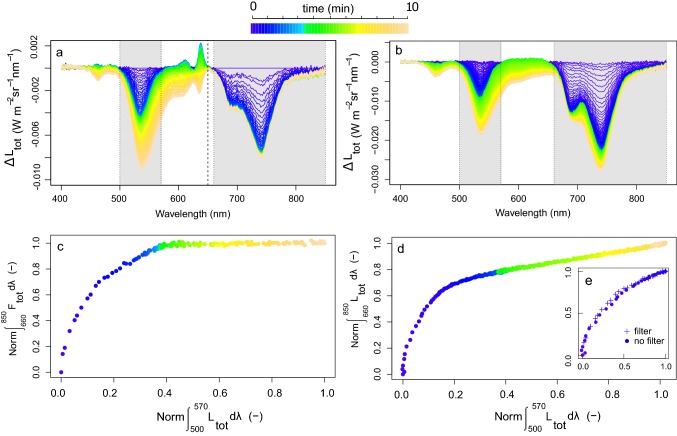
Total radiance changes (Δ*L*_tot_) for a *J. regia* leaf measured according protocol 1, i.e. with filter (**a**) and according protocol 2, i.e. without filter (**b**) the 650-nm cut-off filter during a 10-min dark-to-high light transient. Δ*L*_tot_ values for the spectral ranges 500–570 and 660–850 nm (marked as grey shades) were integrated and normalized with the integral of the total change at the corresponding spectral ranges during *t* = 0–10 min with (**c**) and without (**d**) filter, and additionally for the period *t* = 0–2 min, in which only fluorescence coupled radiance changes took place (**e**)

In accordance with the previous observations, we observed identical fast and slow absorbance changes for *J. regia* leaves with a consistent spectral action feature. Measuring the optical properties with (protocol 1) and without filter (protocol 2) allowed observing the spectral changes beyond 620 nm, which are composed of both absorbance and *F* changes (Fig. 10). When using the filter, the measurements reconfirmed that fast absorbance changes, in immediate response to the ΔpH, coincide with strong *F* quenching, while slow absorbance changes show no further additional *F* quenching (Fig. 10a), as demonstrated before (Fig. 7d). This effect is better seen by plotting the spectral integrals of the two spectral intervals, i.e. 500–570 and 660–850 nm (Fig. 10c) where *F* varies together with the *L*_tot_ at the beginning (*t* = 0–3 min) of the transient (blue dots), but then it reaches stability, whereas *L*_tot_ (500–570 nm) keeps on evolving. Figure 10d shows the same, a similar trend when the filter is not used, but now the integral in the NIR captures both contributions, i.e. emitted *F* and scattered radiance; as a result, *L*_tot_ in the NIR radiance keeps evolving after *t* = 3 min. To assure that no significant quick scattering changes are detected beyond 650 nm (which cannot be observed when using the filter), it was tested that for both cases the F quenching was happening at the same relative speed. This test ensures that no further significant fast absorbance or scattering changes could be detected beyond 650 nm, which cannot be observed when using the filter. The result showing the same relative *F*-quenching speed for the 500–570 nm integral in both cases (Fig. 10e) suggests that all fast spectral changes > 650 nm can be, in the unfiltered case, attributed to *F* quenching only. On the contrary, the slow absorbance changes (*t* = 4–10 min) did not co-occur with further *F* quenching (Fig. 10c). All additional spectral changes beyond 650 nm show a perfect linear relationship with the VIS spectral changes, suggesting that they belong to the same process.

**Fig. 11:**
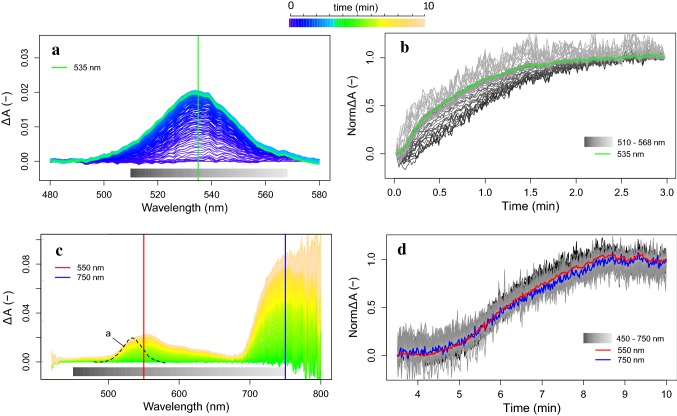
Absorbance changes (Δ*A*) during a 10-min high-intensity illumination (1283 µmol m^−2^ s^−1^) exposure of a dark-adapted *J. regia* leaf, split between the fast (*t* = 0–3 min) Car conversion (**a**) and slow (*t* = 3–10 min) conformational changes (**c**), in both cases referenced by substraction of the absorbance spectrum at the beginning of each phase. The dynamic Δ*A* normalized for the total change of each phase is plotted in time for each phase and each wavelength (marked by grey scales in **a** and **c**), representing the kinetical dynamic of each process (**b**, **d**). The evolution of the absorption change at the peak wavelengths in the VIS–NIR spectral range are given in colour

**Fig. 12:**
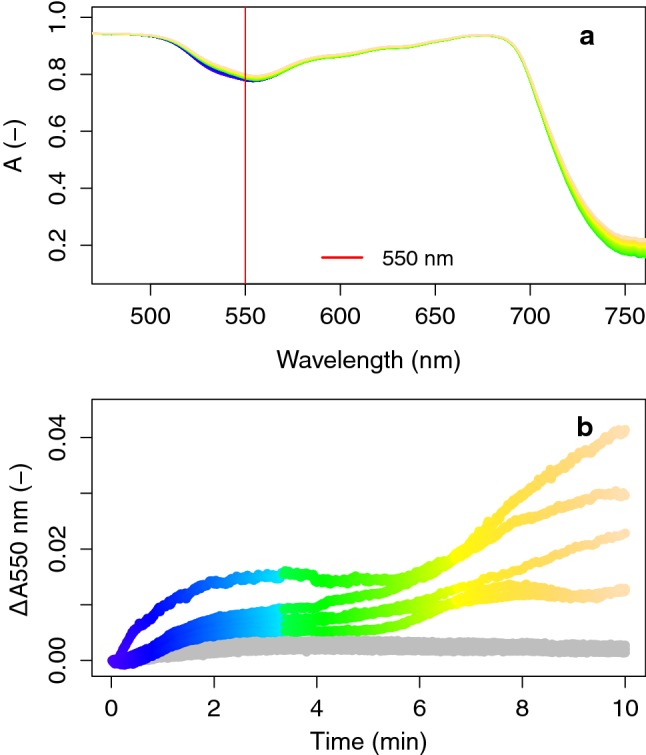
Absorbance profile during the 10-min transient (by colour scale) with shifts caused by the energy-dependent chemical Car conversion (in blue colours) and the slow conformational switch (in green-to-yellow colours) (**a**) and Δ*A* at 550 nm for several leaf transient observations without (grey, *n* = 3) and with (colour scale, *n* = 5) the conformational switch (**b**)

To verify this further, the spectral signatures of both processes with different onset, were further analysed in the spectral ranges of 500–580 nm and 420–850 nm to designate single features based on the wavelength kinetical behaviour. As observed before, the fast Δ*A* is centred around 535 nm (Fig. 11a). The relative cumulative Δ*A* change of this process, calculated for each wavelength in the region 510–570 nm during *t* = 0–3 min, showed a logarithmic trend for all wavelengths (Fig. 11b). Different rates in Δ*A* per wavelength were observed, with *λ* < 535 nm developing Δ*A* at a slower rate than *λ* ~ 560 nm. Analysing the slower absorbance event with onset after 4 min of high light exposure, a smooth spectral feature in the visible and NIR region is revealed (Fig. 11c). Besides the previously shown local green 550-nm absorbance peak, a large Δ*A* was manifested also in the 700–800 nm range, with a wide peak centred around 750 nm. All wavelengths clearly developed at a similar kinetic rate in the entire 450–750 nm range, following a sigmoid function, confirming a single process is dynamically observed (Fig. 11d). This slow but significant process caused a 2% absorbance increase at the 550-nm peak (similar to the quick feature at 535 nm) and a 6–10% absorbance increase around 750 nm. Finally, the absorbance profile during the 10-min transient showing the quick (480–580 nm in Fig. 10a, omitting the *F* changes) and slow (400–800 nm in Fig. 10b) changes is provided in Fig. 12a. Several examinations of the experimental data revealed that the slow absorbance increase was typically observed from *t* = 5 min onward, but probably not always terminated at *t* = 10 min (Fig. 12b).

## Discussion

The detection of spectrally contiguous radiance changes in the VIS–NIR range allowed us to detect, for the first time passively, specific leaf spectral behaviour in the absorbed photosynthetic active radiation (APAR) range with different onsets during strong light adaptation. In contrast to the ultrafast transient absorption spectroscopy of isolated chloroplasts, pigments, or Lhc units that promote single molecules or complexes to an excited state, slower mechanisms are observed during the NPQ built-up phase. NPQ is a kinetically and mechanistically dynamic process, wherefore typically the short-term regulatory chemical and molecular mechanisms have been studied (Deamer et al. 1967; Heber 1969; Bassi and Caffarri 2000; Moya et al. 2001; Ruban et al. 2007, 2012; Johnson et al. 2009a; Johnson and Ruban 2014; Krüger et al. 2014b). It is well understood that two main quick NPQ-related absorption mechanisms occur in the short time upon a ΔpH, with structural rearrangements of the Lhcs as a result. One is the VAZ operation with a pigment-based absorbance feature and the second is the quick PsbS-induced conformational changes or the detachment of the major antenna complexes from the PSII-LHCII core (Kramer and Sacksteder 1998; Johnson and Ruban 2014). In contrast, the occurrence of the slower induced, so-called energy-independent photoprotection mechanisms (Lambrev et al. 2012) are less understood from in vivo observations. Next, we discuss the observed spectral APAR changes and their relation to the NPQ processes, considering additional processes affecting the scattering behaviour at leaf surface as well.

### Fast carotenoid conversions and membrane re-organizations

Carotenoids are known to absorb light at similar wavelengths, with slight spectral shifts and peak positions, due to the conjugation length of the Car, the conformation of the pigment–protein complex (e.g. twisting) and the local environment (e.g. polarity of the medium) (Polívka and Sundström 2004; Polívka and Frank 2016). The S0–S2 transition of the different carotenoids is strongly allowed and visible as the three-peak absorption in the 400–500 nm range in solution. In vivo, protein-bound carotenoids show a red-shift in the absorption spectrum due to the dispersion interactions of the carotenoid with the protein (Polívka and Frank 2016). Due to this absorbance shift, the chemical conversion of the VAZ carotenoids in vivo is observed as an absorbance change in the 500–570 nm region with maximum around 531 nm (Gamon et al. 1990; Peñuelas et al. 1995). The linear correlation between the de-epoxidation state (DEPS) of the VAZ cycle and the reflectance decrease at 531 nm during the first minutes is well known from light transients on intact leaves, as indicated by an accumulation of both A and Z (Bilger et al. 1989; Gamon et al. 1990; Gamon and Surfus 1999; Peguero-Pina et al. 2013). The chemical VAZ conversion observed in this region is hereby exploited for computation of the Photochemical Reflectance Index, i.e. (R531–R570)/(R531 + R570) (Gamon et al. 1990, 1992; Peñuelas et al. 1995). Commonly used in remote sensing applications as indicator for the DEPS, PRI serves as a proxy for the energy or ΔpH-dependent NPQ of vegetation at the leaf and, after required corrections, also at the canopy level. However, other scattering and absorbance shifts than those purely caused by the VAZ chemical conversion are described for the quick phase (Kramer and Crofts 1996; Johnson and Ruban 2014), which may interfere with the concept of a two-band Index (Van Wittenberghe et al. 2018). As a result, and due to the lack of normalization for the pigment pool (Ripullone et al. 2011), the functional link between PRI and DEPS is not always a straightforward relationship, even at the leaf level.

In the short term during strong *F* quenching, we observed several phenomena in the 500–570 nm region with different onsets (Fig. 4a–h, dark grey area). In chronological order upon HL, the following events occurred: (1) an instantaneous two-peaked scattering decrease with maxima around 530 and 560 nm (Fig. 4a, b), followed by (2) a phase whereby *F* is rapidly quenched without further spectral changes (Fig. 4c, d), and next (3) the start of a more prominent feature with a peak around 531 nm (Fig. 4e, f). These observations confirm the *F* quenching associated with the proton gradient (qE) involving two separate but interrelated phenomena: qE-quenching itself (ΔpH built-up), and a modulation or amplification of the quenching by A and Z formation (Demmig et al. 1987). The instantaneous scattering in the same spectral region (Fig. 4a, b) is hypothesized as a low lumenal pH-driven binding of free and available Z, prior to a sufficient ΔpH to activate the VAZ cycle. Similar to the first 2-peak feature in the 500–570 nm region, Kramer and Sacksteder (1998) observed an identical Δ*A* in a short time range of 450 ms upon sudden illumination of an intact leaf, followed by a further one-peak absorbance feature (500–570 nm) stabilizing after 20 s. Compared to our measurements, their experiments reached a faster equilibrium of both absorbance changes, which might be attributed to the use of weaker illumination (300 µmol m^−2^ s^−1^), resulting in a smaller Car pool and faster equilibrium to dissipate the lower photon excess. Apart from this, we have not found any further similar shaped spectral evidence upon sudden illumination. Being located in the green spectral region where A and Z absorption appears in vivo (Gamon et al. 1990, 1992; Peñuelas et al. 1995; Gamon and Surfus 1999), a free and sustained Z pool is suggested to be available prior to illumination, binding to the complexes with the onset of lumen acidification. A sustained Z pool can act a membrane stabilizer in vivo in the lipid phase of the thylakoid membrane (Havaux 1998; Havaux and Niyogi 1999) being involved in antioxidant activity (Havaux and Niyogi 1999; Niyogi 1999; Dall’Osto et al. 2010). Low luminal pH, on the other hand, results in the protonation of specific antenna complexes (Dominici et al. 2002), which, synergistically with Z binding, leads to a conformational change (Moya et al. 2001; Dall’Osto et al. 2005, 2010). In addition, it has been recognized that Z-dependent sustained photoprotection, often grouped under the term qI, consists of two components, one sensitive and the other insensitive to the uncouplers, suggesting different mechanisms operating under different conditions and time scales (Ruban and Horton 1995; Dall’Osto et al. 2005; Nilkens et al. 2010; Ruban et al. 2012). As such, this spectral observation (Fig. 4a, b) could find an explanation in the binding of a readily available Z pool, before the proton gradient is sufficient to start the VAZ cycle. However, since several xanthophylls absorb in a similar region, this hypothesis would need to be further confirmed based on pigment analyses before and during a dark-to-HL transient protocol.

After a *F* quenching devoted to the qE-quenching (ΔpH built-up) itself (Fig. 4c, d), Car conversion (VAZ cycle) becomes visible in the 500–570 nm spectral region in the following minute, with further strong *F* quenching (Fig. 4g, h). Bi-directional Δ*L*_*R*_ and Δ*L*_*T*_ measurements show a parallel and consistent decrease for all measurements, supporting a true absorbance increase at these wavelengths, expected for a chromophore conversion. In parallel to this consistent VAZ absorbance feature, co-occurring fast features are observed either manifesting a decreased (Fig. 4) or increased scattering behaviour with peaks located around 460, 615 and 645 (filter-affected) nm (Figs. 3a, 10a). The spectral signature of these changes are consistent with those observed from the light-induced reorganizations (unstacking) of the thylakoid membranes seen by circular dichroism spectroscopy (Garab 2014). These reversible changes in the organization of the thylakoid membranes occur on the time scale of seconds and minutes depicting the quick disassembly of the multilamellar structures (Garab 2014). Such fast structural response is given the fact that the electrostatic interactions between adjacent lamellae are generally weak (Garab et al. 1991) providing a generally low structural stability compared to e.g. the electrostatic interactions between the complexes themselves (Garab 2014). The physiological significance of this fast membrane flexibility is not fully understood yet, but unstacking or a light-induced increase in the interthylakoidal space would facilitate the mobility of the complexes, required e.g. for the operation of the PSII repair mechanism (Yamamoto et al. 2008). A dip around 615 nm corresponding to the Chl Q_x_ state and decrease of the 675 nm region (here observed at 645 nm) have been identified for quenched states of isolated membranes or isolated LHCII (Ruban et al. 2007). The transient development of these spectral features shown here, illustrating the structural flexibility of the thylakoid membranes in vivo, operates within the same time scale as the VAZ cycle. However, in contrast to the VAZ absorption, scattering changes due to membrane reorganization may not always occur (e.g. Fig. 10b), or show opposite scattering behaviour (Figs. 3-left vs. 4), indicating both processes are not directly related to each other.

### Fluorescence quenching and re-absorption during fast pigment bed changes

Major *F* quenching is seen during the first 3–5 min upon strong illumination, when Car conversion and membrane reorganizations were observed (Fig. 5). During *F* quenching, we observed a small rapid increase in the F687/F740 ratio, followed by a stronger and general decrease the following minutes (Fig. 5d). Possible causes for a *F* peak ratio here, are (i) a change in the relative share of PSI versus PSII *F* emission i.e. due to short-term alternations of the relative PSI-PSII antenna sizes, and/or (ii) an enhanced re-absorption of the red peak (F687) by Chl a, and/or (iii) new fluorescing components of the PSII antenna during NPQ (Miloslavina et al. 2008; Lambrev et al. 2010). Since state transitions between PSI and PSII, balancing light absorption when light quality changes (Niyogi 1999; Xu et al. 2015), are primarily linked to low light conditions, they are considered less important under HL. Lambrev et al. (2010) took a detailed investigation on the NPQ-associated spectral changes in the *F* spectra of *Arabidopsis* (*Arabidopsis thaliana*) measured at room temperature and at 77 K and found for both set-ups a *F* decrease in the red and a *F* increase in the far-red range (> 710 nm), with the first effect larger at room temperature. Mutant analysis showed moreover that the presence or absence of PsbS had a strong impact on the FR changes but not on the red changes, concluding these changes were related to distinct NPQ processes. Interestingly, when Lhcs form aggregates, or higher orders oligomers, they exhibit a relatively enhanced *F* in the far-red region (Miloslavina et al. 2008). Formation of a Chl–Chl charge transfer complex was suggested to be the underlaying cause of this differential *F* quenching (Miloslavina et al. 2008). Formation of such complexes, as a result of Chl aggregation when two Chl molecules interact at a intermolecular separation less than a critical distance of 12 Å (Beddard and Porter 1976), is suggested to play a role in qE and the source for the proposal of exclusively Chl-based quenching (Crofts and Yerkes 1994; Horton et al. 1996; Krüger and van Grondelle 2017). Here, we observed a significant increase in absorption at 615 and 645 nm (Fig. 4i, j) due to membrane reorganizations in the short term. They, however, do not appear as broad absorption bands as would be expected due to strong inter-pigment coupling for charge transfer states (Krüger and van Grondelle 2017). Their appearance in the red region (only observable until 650 nm due to the filter) could, however, explain an enhanced red *F* re-absorption by Chl a causing the decrease of the F687/F740 ratio during quick arrangement of the membrane upon excessive light.

### Slowly induced conformational pigment-protein changes in the VIS–NIR

Following the fast carotenoid conversion and membrane reorganizations, a slower broad VIS–NIR absorbance increase was observed for both species, identified as one dynamical process (Fig. 11c, d). The action feature characterized by a consistent green and far-red absorbance increase intuitively contradicts with a light avoidance strategy. In what follows next, we intend to aggregate the arguments and provide an evidence-based framework, which help to understand the slow manifestation of this mechanism which has not been measured before in the view of light excess. Hereby, we discuss certain confusing phenomena (e.g. chloroplast motion) and instrumental artefacts that could be (mis)understood from the given observations:

i. *Instrumental reliability and set-up* Any instrumental or set-up-induced artefact constant in time which would alter the given radiance flux (Supplementary Material S1) is accounted for, diminished or would be nullified calculating dynamical spectral radiance flux changes. Dynamical artefacts such as possible instrumental drift are not compatible or realistic with our observations (up to 8% *L*_tot_ signal change in the NIR within 10 min), given the specific spectral nature and temporal behaviour of the feature. No LED instability was observed, verified by the surface radiance *L*_S_ before and after each transient, and would be seen as an overall (irregular) increase or decrease in *L*_up_ or *L*_dw_, inconsistent with the spectral feature;
ii. *Absorbance increase* At the intact leaf level, in-path scattering changes are neutralized by the multiple scattering inside the leaf, wherefore, the Δ*A* is given only by specific absorbance changes. This allows us to observe specific spectral patterns for leaf-internal fast Car conversion, membrane reorganizations or slow chloroplast motion at a high temporal resolution. A decrease in both consistent forward and backscattered diffuse light during these slow observations indicates a true specific absorbance increase (Fig. 12a, b), whereby transmitted light changes are stronger affected (Figs. 7, 8). An overall absorbance increase contradicts to slow chloroplast motion, involving a spectrally broad Δ*A* decrease demonstrating a light-avoidance strategy (Deamer et al. 1967; Brugnoli and Björkman 1992; Dutta et al. 2017). The slow Δ*A* increase is further spectrally distinctive from chloroplast motion due to the lack of strong peak features in the 400–500 nm region (Fig. 11c) and the absence of any *F* decrease (Fig. 10c). A specific absorbance increase also further contradicts to the absorbance changes in the VIS–NIR during progressive desiccation (water absorbs in the NIR), which would be manifested as an absorbance decrease in the NIR (Peñuelas and Inoue 1999);
iii. *Light-harvesting antenna framework* Given the broad nature and specific shape of the absorbance increase, any chemical change leading to such a new absorbance component is however not considered realistic. On the other hand, red-shifted spectra of Car and Chl absorptions as well as for *F* bands have been commonly observed under high light exposure during qE formation of isolated Lhcs (Bode et al. 2009; Liao et al. 2010), or for Chl *F* spectra (Johnson and Ruban 2009) and in vivo (Bode et al. 2009; Lambrev et al. 2010). The explanation for these absorbance shifts can be found in the fact that protein-embedded pigments are known to serve as effective probes of conformational changes providing structural flexibility, affecting their spectral properties (Scholes et al. 2011; Krüger and van Grondelle 2017). Hereby, the protein serves as the scaffold that controls the organization of the pigments in the Lhcs whereby strong intra-pigment interactions can considerably increase the sensitivity of the pigments to the local environment (Novoderezhkin et al. 2007). In response to light excess, the local environment of the Lhcs is shown to be heterogeneous dielectric, profoundly tuning the energy transfer between pigments, caused by changes is the relative orientations of the pigments (Curutchet et al. 2011). The formation of the earlier mentioned red-shifted bands of Car and Chl has been correlated to the excitonic coupling between the S1 state of carotenoids and the lower states of Chl whereby, surprisingly, and increased Car → Chl as well as Chl → Car energy transfers were observed (Bode et al. 2009). Such interactions can occur when the two state energies are similar and there is as significant electronic coupling, which has been confirmed to apply for the Car S1 state and the lowest excited singlet states of Chl a, Chl Q_y_ (Bode et al. 2009). Under these conditions, the coupling creates two new states a least partially delocalized over both pigments, leading to a collective excitation known as exciton. Exciton states seem to be present to some extent in all photosynthetic Lhcs (Scholes and Fleming 2006), and can shift spectra substantially, leading to quite marked spectral inhomogeneity (Müh et al. 2010). These observations have however only been observed in the fast response to light and linked to the PsbS-dependent conformational change during qE;
iv. *Timing* The onset of the absorbance change takes place after 3–5 min of constant HL exposure, when the fast changes have terminated. This behavioural time pattern seen for all observations (Fig. 12b) suggests the spectral changes might relate to the preceding fast changes, e.g. the formation and binding of Z, indirectly and invariably observed during the first minutes (Fig. 11a, b). The current acceptance of Z being the allosteric regulator for the slow conformational changes (Ruban et al. 2007; Ilioaia et al. 2011), might suggest the observed spectral changes relate to a structural modification induced after binding of Z (see point vi), although further triggers would apparently be needed. At the Lhc level, Z-dependent quenching is observed as a more slowly induced reorganization compared to the fast PsbS-dependent conformational changes (Holzwarth et al. 2009; Nilkens et al. 2010; Lambrev et al. 2012). The latter were, however, not observed;
v. *Kinetics* The evolution of the spectral feature shows a slowly developing kinetic behaviour (Δ*t* = 7 min) with strong sigmoidicity (Fig. 11d). Sigmoidal kinetic profiles are the result of enzymes that demonstrate positive cooperative (allosteric) binding, i.e. binding of a ligand at one site of the macromolecule increasing the enzyme’s affinity for another ligand at a site different from the other site (cf. Hill equation). During qE, such kinetical behaviour has been observed (Horton et al. 2000). For enzymatic reactions with multiple substrate-binding sites such as the Lhcs, this increased affinity for the substrate can cause a rapid and coordinated increase when a certain concentration of ligands is bound to the macromolecule. The protein-embedded pigments of the Lhcs not only interact with one another but also with the protein itself, and, hence, protein structural fluctuations (e.g. as a result of an enzymatic binding reaction) may significantly alter their spectroscopic and light-harvesting properties by tuning the pigment’s transition energy (see point iii). Our observations showing the simultaneous spectral dynamic according to this slow sigmoid kinetic suggest the interaction between the protein scaffold and the pigments (see point vi) plays an important role. It should be mentioned, that our monitoring time was likely often too short to monitor the slowly induced conformational change being built-up until the final state (Fig. 12b), except the cases shown in Figs. 7–11 showing a steady-state or saturation in the signal response. Hence, a longer observation time is advisable. This was also recently corroborated by 20-min light transients performed on *Fagus sylvatica* L. leaves, showing identical strong slow absorbance increases, observable from the reflectance signal (Van Wittenberghe et al. 2018);
vi. *Shape and peak locations* The observed smooth absorbance change shows widened absorbance features around 550 and 750 nm, which suggests involvement of both red-shifted Car and Chl (point iii). Other proteins or molecules may absorb in the NIR (e.g. plastocyanin, P700 +), however there is no experimental evidence they show any (slow) absorbance dynamics upon NPQ induction. Figures 9b and 11c illustrate for both *M. alba* and *J. regia* the similar magnitude (i.e. 2%) in Δ*A* and shape between the VAZ feature and the slow feature, seemingly affected by a 20-nm spectral shift and very large band broadening. The conformational motions of pigment-protein complexes perturbing the site energies can be considered an explanation for these effects (see point iii). As mentioned, the energies of excitonic transitions are determined by the combined action of exciton shifts (causing homogeneous broadening of the main absorption peak together with the appearance of vibrational wings) and reorganization shifts (causing inhomogeneous broadening due to disorder) (Novoderezhkin et al. 2007; Krüger and van Grondelle 2017). The 550-nm feature indeed shows a strong vibrational tail until 680 nm. A most plausible explanation is that the antenna complexes where the Z was earlier bound are further involved in the slow structural transition, observed from (1) the shifted peak location, (2) the equal magnitude, and (3) the specific timing, as the slow absorbance changes were only seen after the VAZ cycle terminated (Fig. 12b). Interestingly, the feature extends to the NIR and kinetically couples with a wide peak at 750 nm with an approx. 50 nm half bandwidth (Fig. 11c). These observations are similar to the red-shifted behaviour observed during qE, explained by exciton mixing between Car S1 state and the Chl Q_y_ states at sub-leaf level (point iii), suggesting a similar mechanism. Low-energy Chls, also called red forms, have been observed in quenched states of individual complexes (Miloslavina et al. 2008; Wientjes et al. 2012), shifting the *F* emission spectra in vitro. Differences in red absorbance > 700 nm from circular dichroism spectra also illustrated these red forms (Morosinotto et al. 2003). Moreover, the spectral shift and large homogenous broadening of these red forms were explained due to mixing with a CT state (Romero et al. 2009). Our in vivo observation of a very broad continuous absorbance feature over these specific wavelengths may not necessarily related to the same absorbance shift described during qE or PsbS-dependent conformational changes, but seem to indicate an analogous behaviour of strong coupling between the Car (suggestively Z) and Chl Q_y_ states.

In vivo, regulated photoprotection has been commonly understood from quick 1Chl* excitation energy quenching or the *F* quenching pathway only (cf. PAM-NPQ). However, the consensus is that non-photochemical quenching involves protein conformational changes, in the case of the fast changes, re-distributing the energy levels of particular pigments in the Lhcs and creating energy traps (Krüger and van Grondelle 2017). Two types of energy traps are under the loop: (1) a low-lying Car S1 state and, (2) a CT state either formed by Chl–Chl or Chl–Car interaction (Krüger and van Grondelle 2017). Experimental evidence for these formations has been given mostly by in vitro observations at sub-leaf level. However, at the intact leaf level specific absorbance changes resulting from the whole ensemble of internal structural rearrangements allows us to observe specific spectral dynamics and their dynamical and sequential behaviour. The strong absorbance increase observed according a slow sigmoid dynamic is hereby suggested to find its origin similar as the one of the known fast low-energy red-shifted components in the absorption spectra of PSII peripheral antenna complexes during short-term fast conformational changes (Ruban et al. 1993; Miloslavina et al. 2008; Bode et al. 2009; Johnson et al. 2009a, b), suggesting these pathways for energy quenching. Hence, a low-energy (long-wavelength) shifted feature indicating a strong coupling between Car (peak at 550 nm) and Chl (peak at 750 nm) states is proposed as possible explanation for our observations. Fast broadband shifted features during qE were not observed for the two species, indicating the absence of these PsbS-dependent structural changes. This could be considered in agreement with the models suggesting two independent NPQ sites (peripherical and core antenna) operating independently and allowing a fast and a slow regulation response to HL in order to avoid severe overshoots as well as undershoots (Holzwarth et al. 2009; Holzwarth and Jahns 2014). Moreover, although being tested under equal illumination and temperature conditions, the slowly induced absorbance shift did not take place systematically in all leaves tested after the occurrence of Z formation (Fig. 12b). A possible explanation we seek in this context is that the activation energy, required to arrive to this further state, is quite high. Protein motion can be described as transitions between different conformational sub-states, whereby the transition of the protein complex requires additional energy to cross barriers in the context of the conformational-energy landscape model (Krüger et al. 2014a). Hereby, the transition energies of the pigments and shapes of their absorption and fluorescence spectra, together with how the molecules are assembled, decide the energy landscape (Scholes et al. 2011). Depending on the pigment pool available and pigment bed organization formed by previous light conditions (even after 2 h darkness), the amount of energy needed to activate a further conformational change might have possibly varied between leaf samples. This probably makes the switch towards a further conformational state in the chain of conformational disorder within a short transient time of 10 min a relatively unpredictable event. Further experimental evidence from systematic experiments is needed to confirm this order of events for more species and to investigate the link between available pigment pools and the absorption dynamics upon light excess. Careful selection of species and growing conditions and the possible use of specific mutant species may help to further disentangle the absorbance dynamics related to the regulated and structure-based energy distribution and possible energy trapping of light excess.

## Conclusions

Spectral dynamics of bi-directional diffusively scattered light at the leaf level upon illumination excess allow us to observe in vivo a complexity of structural adjustments at different hierarchical levels of the photosynthetic machinery. Fast dynamics involve a consistent VAZ conversion feature, often in parallel with more irregular membrane re-organizations within the first minutes, when 1Chl* or *F* quenching takes place. VAZ conversion is hereby seen as an absorbance increase in the 500–570 nm region from both leaf sides due to a change in chromophore composition. As a slower response, a further and major VIS–NIR absorbance feature is observed and argued to be a shift due to a slow-induced pigment-protein conformational switch, with no *F* quenching involved. This spectral feature is clearly distinct from the chloroplast motion photo-avoidance strategy that happens also slowly but is manifested as an overall absorbance decrease. The broadened and very significant green–red (~ 550 nm) and near-infrared (~ 750 nm) peaks in this smooth feature are suggested to be a low-energy shifted re-distribution of absorption bands of, respectively, Car and Chl, showing a strong exciton coupling. The biophysical cause of these changes is further argued to be found in the pigment-protein dynamics whereby spectral properties of the pigment bed are fine-tuned due to modified interactions between the chromophores. Yet, these new insights need to be corroborated by further well-designed experiments to confirm the observation of non-photochemical energy quenching identified as a structure-based energy dissipation mechanism detectable in vivo, over the full PAR spectrum and beyond 700 nm. Certainly, the identification and unravelling of the dynamical spectral features detected through intact leaf spectroscopy seems to be a very promising direction in the further understanding of pigment–protein complex dynamics in their native environment. Such developments will allow us to observe the APAR energy distribution at a higher detail, i.e. with a harvesting or a dissipation fate, and to improve the monitoring of in vivo plant functioning from remote VIS–NIR hyperspectral observations.

## Electronic supplementary material

Below is the link to the electronic supplementary material.

Supplementary material 1 (DOCX 14 kb)
