# Supplementary material for: In vivo photoprotection mechanisms observed from leaf spectral absorbance changes showing VIS–NIR slow-induced conformational pigment bed changes
Source: Photosynth Res. 2019 Sep 20;142(3):283–305. doi: 10.1007/s11120-019-00664-3 (PMC6874624; doi:10.1007/s11120-019-00664-3)
Supplement: Supplementary file 1 — Supplementary material 1 (DOCX 14 kb) [file 11120_2019_664_MOESM1_ESM.docx]

**Supplementary material S1**. Possible artefacts that were considered and mitigated by the leaf clip design and experimental set-up.

**Light stability**. The LED lamp was powered by a stabilized power source, removing any drift in intensity due to voltage or current oscillations. The LED was actively cooled to keep its working temperature constant and avoid illumination drift due to thermal changes. LED stability was moreover checked by surface radiance L_S_ measurements before and after each transient.

**Stray light**. The experiment took place in open space within a spectrally dark room (reflectance lower than 5% in the full optical spectrum (from 350 to 2500 nm) and fixed on an optical bench that ensured stability and alignment. Objects and instruments were kept hidden from view to avoid unwanted reflections on the sample. Structures and leaf clip interior were also black. There were no moving parts that could alter how the illumination reached the sample during the experiments that were conducted in darkness. A measurement with all instruments on but the lights off reported no signal being collected by the spectrometers to the level of their sensitivity.

**Background contribution**. The sample was fixed in the clip with a square frame, with no substrate below or above the sample (not even transparent glass). The light from the sample was collected by fiber optics to keep a low profile of possible scattered light. The table below was dark and far (at least 30 cm from the leaf) to minimize light reflected from the table surface reach the sample again.

**Radiometric calibration**. Spectrometers were radiometrically calibrated to a standard and non-linear response was corrected, thus reporting the measurements in physical units.

**Dark noise in the spectrometers** (signal reported by the sensors under the absence of light). Dark noise was characterized before and after the measurements with the same operation configuration than used for the measurements. A large enough dark noise samples were acquired to properly obtain the mean response, and also to evaluate possible drifts. Dark noise was removed from the measurements to obtain the signal produced by light.

**Filter transmittance**. A slight amount of light beyond the cut-off wavelength can pass the filter, especially close to the cut-off wavelength. For this, the incoming light (E(λ)) is multiplied by a previously determined filter transmittance factor τ(λ) calculated for the region 650-800 nm as τ(λ) =E_filter_ (λ)/ E (λ). Hence, the minor contamination of the light transmitted by the filter and subsequently scattered by the leaf is calculated as τ(λ)* E (λ)* R(λ) and τ(λ)* E (λ)* T(λ) for respectively L_up_(λ) and L_dw_(λ).
